# First-In-Class Inhibitors Targeting the Interaction between Bacterial RNA Polymerase and Sigma Initiation Factor Affect the Viability and Toxin Release of *Streptococcus pneumoniae*

**DOI:** 10.3390/molecules24162902

**Published:** 2019-08-09

**Authors:** Jiqing Ye, Adrian Jun Chu, Lin Lin, Xiao Yang, Cong Ma

**Affiliations:** 1 State Key Laboratory of Chemical Biology and Drug Discovery, Department of Applied Biology and Chemical Technology, The Hong Kong Polytechnic University, Kowloon, Hong Kong; 2 Department of Microbiology, The Chinese University of Hong Kong, Prince of Wales Hospital, Shatin, Hong Kong

**Keywords:** RNA polymerase, sigma factor, transcription, inhibitor, antimicrobial discovery

## Abstract

Novel antimicrobial classes are in desperate need for clinical management of infections caused by increasingly prevalent multi-drug resistant pathogens. The protein-protein interaction between bacterial RNA polymerase (RNAP) and the housekeeping sigma initiation factor is essential to transcription and bacterial viability. It also presents a potential target for antimicrobial discovery, for which a hit compound (**C3**) was previously identified from a pharmacophore model-based *in silico* screen. In this study, the hit compound was experimentally assessed with some rationally designed derivatives for the antimicrobial activities, in particular against *Streptococcus pneumonia*e and other pathogens. One compound, **C3-005**, shows dramatically improved activity against pneumococci compared to **C3**. **C3-005** also attenuates *S. pneumoniae* toxin production more strongly than existing classes of antibiotics tested. Here we demonstrate a newly validated antimicrobial agent to address an overlooked target in the hit-to-lead process, which may pave the way for further antimicrobial development.

## 1. Introduction

Infections by multi-drug resistant (MDR) bacteria (*superbugs*) have become an increasingly significant health burden worldwide [1]. *S. pneumoniae* causes serious febrile illnesses such as pneumonia, septicemia, and meningitis, but its susceptibility to existing classes of antimicrobials is also on a decline [2]. *S. pneumoniae* mediates disease through a wide range of well-characterized virulence factors such as pneumolysin to facilitate colonization, nutrient scavenging, and immunoevasion [3]. Commonly-used bacteriolytic antimicrobials such as β-lactams have often been criticized for their role in the undesired elevation of *S. pneumoniae* toxin levels into host environments and affecting the treatment outcome of *S. pneumoniae* infections [4].

**Figure 1:**
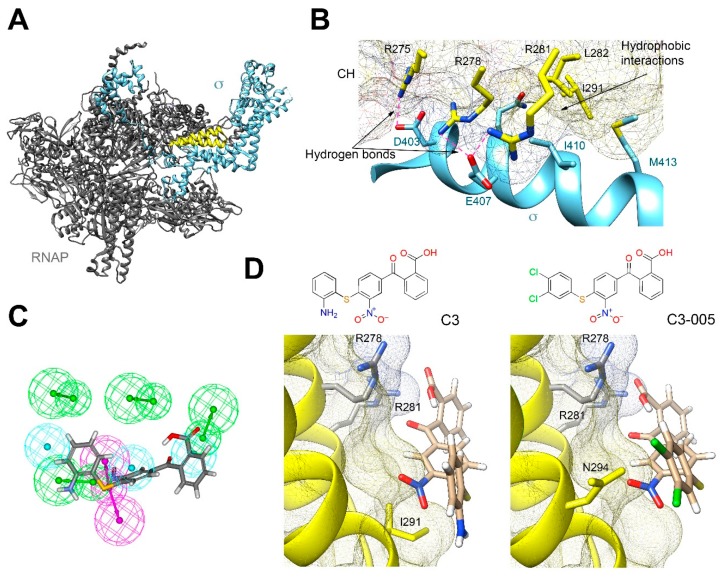
Inhibitors of RNAP-σ interaction. (**A**) The crystal structure of *E. coli* RNAP holoenzyme (PDB: 4LJZ; 27) with the RNA polymerase core enzyme colored in gray, the CH region in yellow and σ^70^ in blue. (**B**) The interaction between RNAP CH region (surface in mesh) and the N-terminal domain of σ^70^ (blue helix) with key amino acid residues labeled. (**C**) **C3** docked in the pharmacophore model. Green spheres, H-bond acceptors; pink spheres, H-bond donor; cyan spheres, hydrophobic groups. (**D**) The docking model **C3** (left) and **C3-005** (right) with the CH region in yellow helix and mesh surface.

The identification of unprecedented targets is crucial to the discovery of novel antimicrobial agents for treatment against infections caused by *superbugs*. Amongst the antimicrobial classes, agents specifically targeting bacterial transcription are under-represented with only rifamycins and fidaxomicin in current clinical use [5]. For bacterial transcription, the well-characterized bacterial RNAP core enzyme is responsible for binding to DNA template and RNA synthesis. This process is facilitated by the formation of a crucial RNAP holoenzyme by the core enzyme with a σ factor (Figure 1A), which is responsible for the initiation of promoter-dependent transcription [6]. The essential house-keeping σ factor is referred to as σ^70^ (in *Escherichia coli*) or σ^A^ (in *Bacillus subtilis*) and one of the major binding sites occurs between the highly conserved clamp-helix (CH) region of RNAP and the N-terminal domain of σ (Figure 1B) [7]. These sigma factors have been exploited for upregulation or inhibition of bacterial transcription through engineered or synthetic modulators [8].

**Figure 2:**
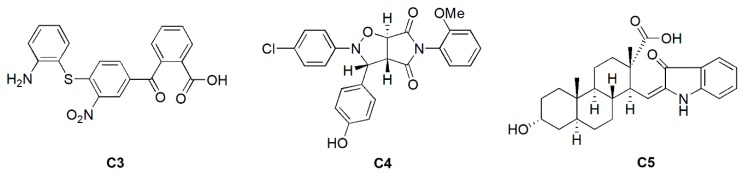
Three hit compounds **C3**, **C4**, and **C5** previously identified by *in silico* screening.

The interaction between RNAP and σ factors has been considered a target for novel antimicrobial discovery [9,10,11,12,13] as opposed to other inhibitors which target RNAP enzyme activities (such as rifampicin binding near the active site, lipiarmycin leading to allosteric inhibition of template DNA binding, myxopyronin and squaramides blocking the switch region of the RNAP clamp open-close) [5]. Previously, by rational design and pharmacophore model-based *in silico* screening, we have identified three chemical compounds (Figure 2) that inhibit bacterial RNAP-σ interaction by binding to the CH region of RNAP [14]. One of the three compounds (**C5**), composed of a steroidal ABC tricyclic ring and an indolone moiety which commonly appear in natural products, was chosen for characterization. **C5** was shown to inhibit RNAP-σ interaction in an ELISA-based assay as well as an *in vitro* transcription assay [14]. **C5** demonstrated mild bacterial growth inhibition against both Gram-positive *Staphylococcus aureus* and Gram-negative *E. coli*. While the mechanism of **C5** was established both at the molecular and cellular level [14], the other two compounds, **C3** and **C4**, require further studies for their antimicrobial potential.

## 2. Results and Discussion

### 2.1. Docking Study of **C3** and Its Antimicrobial Activity

We are particularly interested in **C3** as it is a small molecule with drug-like properties predicted by Discovery Studio 2016 (Biovia, San Diego, California, United States). The substituted benzene rings can be easily modified and are suitable for studying the structure-activity relationship and validating our previously established pharmacophore models (Figure 1C). Nevertheless, modifications may be made to improve the inhibitory and antimicrobial activity of C3. As shown in the docking model (Figure 1D, left), **C3** fits into the pharmacophore model using the right benzoic acid to form an ionic bond as the key anchor to R278 or R281 of *E. coli* RNAP CH, while the left substituted benzene ring may form interactions with I291 of RNAP CH by van der Waals forces, which is appropriate for an initial modification to probe the interaction with RNAP CH and identify a lead compound for further studies.

The antimicrobial activities of **C3** were first tested to determine the minimum inhibitory concentration (MIC) in accordance with the guidelines published by the CLSI using six bacterial species from the most recent “WHO priority pathogens list for guiding R&D of new antibiotics” consisting of three Gram-positive and three Gram-negative bacteria: *Enterococcus faecalis*, *S. aureus*, *S. pneumoniae*, *Acinetobacter baumannii*, *Pseudomonas aeruginosa*, and *Enterobacter* spp. [15]. **C3** shows very mild antimicrobial activity (MIC 256 μg/mL) against *S. pneumoniae* ATCC 49619 (Table 1).

### 2.2. Molecular Mechanism of **C3** by Inhibiting the Protein-Protein Interaction between RNAP CH-σ

We then confirmed the mechanism of **C3** at the molecular level by assessing the inhibition against the *in vitro* protein-protein interaction (PPI) at the major binding site between RNAP CH region and σ. Previously established split-luciferase assay was employed [16], in which the *B. subtilis* CH region of RNAP (amino acid 220-315) and full-length σ^A^ were each tagged with one of the luciferase complementation fragments. In the absence of inhibitors, the interaction between CH-σfacilitates the reformation of the luciferase indicated by the luminescence released. Reduction of the luminescence signal due to inhibitor treatment reflects the percentage of inhibition of the PPI between CH-σ as compared to the control without inhibitor. As a result, the IC_50_ of **C3** against the PPI between CH-σ at 0.05 μM was measured as 6.40 ± 0.71 μM (Figure S1). This suggested the **C3** compound was able to inhibit CH-σ interaction as designed. The full data set of the assay was illustrated in Table S1. We also measured the percentage of inhibition of **C3** at 10 μM against the PPI between CH-σ, which can be used to facilitate the activity comparison with **C3** derivatives (Table 2) (17).

### 2.3. Antimicrobial and Inhibitory Activities of **C3** Derivatives

**Scheme 1:**
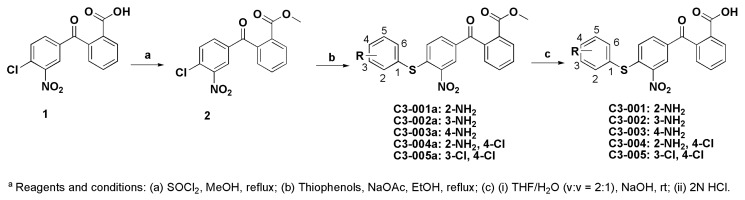
Synthesis of **C3-001–005**.

We proceeded to construct chemical derivatives of **C3** (named **C3-001** in the library) to probe the inhibitory and antimicrobial activities by modification of the position of -NH_2_ on the left benzene ring (Scheme 1). As shown in Table 2, when the amine moved from 2-position of the left benzene ring to 3-position (**C3-002**), both the inhibitory and antimicrobial activity against *S. pneumoniae* improved, which can be explained by the additional interaction with I291 of RNAP CH. As shown in Figure 1D, the 3-position of the left benzene ring of **C3** is closer to I291 than the 2-position. While **C3-003** with 4-NH_2_ gave similar results to **C3-001**, we decided to add one chloride group at 4-position of **C3-001** to form **C3-004**, intending to probe the van der Waals interaction with N294 of RNAP CH. The result showed that **C3-004** maintained the inhibitory activity but improved the antimicrobial activity. The increased antimicrobial activity may be the effect on logP by replacing amine with chloride to improve cell permeability. As the substitution at the 3-position (**C3-002**) was preferred compared to the 2-position (**C3-001**) for improving both the inhibitory and antimicrobial activity, based on the data, we synthesized a 3,4-dichloro compound **C3-005**, which demonstrated a dramatically improved antimicrobial activity (MIC 8 μg/mL; Table 1 and Table 2) and similar inhibitory activity against RNAP CH and σ to **C3-002** (Table 2).

### 2.4. Antimicrobial Activity of **C3-005** against Representative Gram-Positive Bacterial Pathogens

We expanded the antimicrobial activity testing of the **C3** derivatives to a selected panel of clinically relevant pathogens, as shown above. In our screening, the Gram-positive bacteria were generally more responsive to the anti-σ compound than the Gram-negative pathogens (Table 1)–a result normally attributable to altered permeability and efficient efflux mechanisms. Nonetheless, this warrants further investigation and synthesis in the near future.

Since *S. pneumoniae* showed particular susceptibility to the compound series, we extended our antimicrobial activity testing to groups A and B *Streptococci*: *Streptococcus pyogenes* (Group A *Streptococcus*, GAS) causing strep throat, localized skin infection and necrotizing fasciitis [17], and; *Streptococcus agalactiae* (Group B *Streptococcus*, GBS) causing neonatal infections [18]; as well as clinically significant Gram-positive pathogens *Staphylococcus epidermidis* and *Staphylococcus saprophyticus*. The MIC of **C3-005** against GAS and GBS strains (MIC 16 μg/mL) and other Gram-positive pathogens was at a similar level to that of *S. pneumoniae* (Table 3).

### 2.5. Time-Kill Kinetics

**Figure 3:**
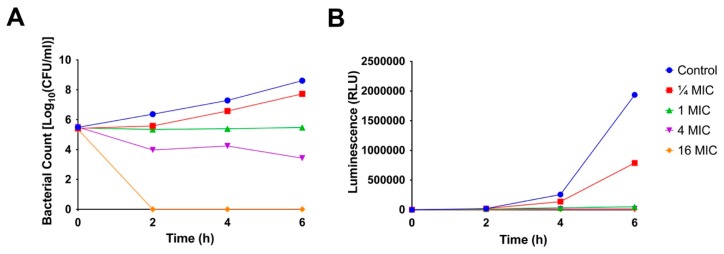
The effect of **C3-005** on (**A**) the time kill kinetic and (**B**) ATP production of *S. pneumoniae* cells when challenged at ¼×, 1×, 4× and 16× MIC.

The time-kill kinetics study reflects the effect of antimicrobial agents to the growth of bacteria at diverse concentrations over time. We constructed time-kill curves by administering **C3-005** to *S. pneumoniae* at various concentrations and to assess its *in vitro* antimicrobial activity. The *S. pneumoniae* cells were grown in liquid culture with agitation and 5% CO_2_ pursuant to the CLSI guidelines [15]. **C3-005** was bacteriostatic at 1 × MIC, while at 4 × MIC a decrease in CFU counts could be observed (Figure 3A). At 16 × MIC eradication of colonies below the level of detection (200 CFU/mL) was achieved from 2 h onwards (Figure 3A). This suggested that **C3-005** acts primarily in a bacteriostatic manner at lower concentrations but was capable of rapid bactericidal effects (>3-fold log_10_ decrease) at higher concentrations.

### 2.6. ATP Production

The inhibition of vital components central to bacterial metabolism, such as respiratory ATP synthesis, is one of the hallmarks of an effective antibiotic [19]. We therefore monitored the ATP production over time in the presence of **C3-005** at various concentrations in *S. pneumoniae* cells. The same culturing conditions as that of the time-kill assay were followed. The rate of ATP production saw a marked decrease at ¼ × MIC compared to the untreated control, whereas higher concentrations of **C3-005** further arrested cellular respiration (Figure 3B). This trend mirrors the previously reported impact of rifampicin, the leading transcription inhibitor drug, on pathogen respiration, as well as its established correlation with antimicrobial efficacy [20,21].

### 2.7. S. pneumoniae Toxin Secretion

**Figure 4:**
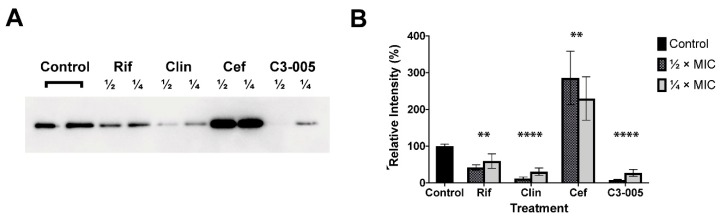
The effect of **C3-005** on the secretion of *S. pneumoniae* pneumolysin. (**A**) Western blot showing pneumolysin levels extracted from culture supernatant following overnight incubation of *S. pneumoniae* with ½ × (checkered bars) and ¼× (light grey bars) MICs of **C3-005**, rifampicin (Rif), clindamycin (Clin), ceftriaxone (Cef), and; (**B**) relative intensity of blotted bands normalized against the drug-free control (solid black bar). One-way ANOVA was used to compare data across the treatment groups. Data was presented in GraphPad Prism style: *p* ≤ 0.05 (*), ≤ 0.01 (**), ≤ 0.001 (***), ≤ 0.0001 (****).

Release of the toxin pneumolysin into the extracellular milieu is a signature virulence factor of *S. pneumoniae* and non-lytic antimicrobials such as macrolides and rifampicin have been shown to repress pneumolysin release at sub-MIC concentrations [4,22,23]. In this study we explored the impact of **C3-005** on *S. pneumoniae* pneumolysin secretion. Adapting from the CLSI guidelines, *S. pneumoniae* cells were cultured overnight in Brain-Heart Infusion (BHI) media at ½× and ¼× of the corresponding pre-determined MICs of **C3-005**, the non-lytic antimicrobial rifampicin, clindamycin, and the bacteriolytic agent ceftriaxone, along with a drug-free control [15]. The cultures were then centrifuged, and the supernatant harvested for Western blot analysis. There were significant differences between Control and ½ x and ¼ x MIC of the treatment groups, Rifampicin (*p* = 0.0018), Clindamycin (*p* < 0.0001), Ceftriaxone (*p* = 0.0026) and **C3-005** (*p* < 0.0001) analyzed by the one-way ANOVA method. The three control drugs performed as previously reported, where rifampicin and clindamycin decreased toxin release while the bacteriolytic antimicrobial agent ceftriaxone drastically promoted the level of pneumolysin [24]. Significant reduction of post-culture toxin levels by **C3-005** was observed over the untreated control, as well as more toxin reduction than rifampicin (Figure 4). The trends were also highly comparable with that of the bacteriostatic lincosamide clindamycin, indicative of growth repression mechanism without the induction of pneumolysin (Figure 4).

### 2.8. Cytotoxicity of **C3-005**

With the best antimicrobial activity among the **C3** derivatives, **C3-005** was subjected to cytotoxicity testing against HepG2 human liver cancer cell line (HB-8065™, the American Type Culture Collection, Manassas, Virginia, United States) and A549 human lung carcinoma cell line (CCL-185™, the American Type Culture Collection, Manassas, Virginia, United States). As shown in Table 4, **C3-005** did not show significant cytotoxicity against the mammalian cell lines compared to the anti-cancer drug cisplatin control, indicating a promising clinical prospect base on the lead optimization of **C3-005**.

## 3. Materials and Methods

### 3.1. Chemistry

Starting materials and regents, unless otherwise stated, were of commercial grade and used without further purification. All reactions were monitored by thin-layer chromatography (TLC) on glass sheets (Silica gel F_254_) which can be visualized under UV light. Flash chromatography was carried out using silica gel (200–300 mesh). ^1^H-NMR (400 MHz) and ^13^C-NMR (100 MHz) spectra were measured on BRUKER AVANCE-III spectrometer with TMS as an internal standard. Chemical shifts are expressed in δ (ppm) and coupling constants (*J*) in Hz. High resolution MS spectra were measured using a QTOF-2 micromass Spectrometer by electron spray ionization. HPLC analysis was performed on an Agilent 1260 HPLC apparatus.

#### 3.1.1. Synthesis of Methyl 2-(4-chloro-3-nitrobenzoyl)benzoate (**2**)

A solution of 2-(4-chloro-3-nitrobenzoyl) benzoic acid **1** (3.057 g, 10 mmol) in MeOH was cooled to 0 °C followed by a dropwise addition of thionyl choloride (0.5 mL). The mixture was refluxed for 24 h. After evaporation of the volatiles, the residue was treated with 5 mL MeOH and stirred at room temperature for 10 min. The precipitate was collected by filtration and dried in vacuum to give compound **2** as a white solid (2.877 g, 90%).

#### 3.1.2. General Procedure for the Synthesis of Compound **C3-001a** and its Derivatives

To a flask was added compound **2** (64 mg, 0.2 mmol), benzenethiol (0.24 mmol), NaOAc (82 mg, 1 mmol.) and EtOH 5 mL. The mixture was heated to reflux for 4h. After cooling to room temperature, the precipitate was collected *via* filtration and washed with appropriate EtOH and water successively, then dried in vacuum to give the titled compounds. Otherwise, water was added and the aqueous layer was extracted by EtOAc. The combined organic layers were dried over Na_2_SO_4_ and purification by column chromatography to provide the titled compounds.

Methyl 2-(4-((2-aminophenyl)thio)-3-nitrobenzoyl)benzoate (**C3-001a**): Yellow solid, 60 mg, 73% yield. ^1^H-NMR (400 MHz, CDCl_3_) δ 8.60 (d, *J* = 1.6 Hz, 1H), 8.10 (d, *J* = 7.7 Hz, 1H), 7.76 (dd, *J* = 8.5, 1.7 Hz, 1H), 7.68 (td, *J* = 7.4, 1.0 Hz, 1H), 7.61 (td, *J* = 7.5, 0.9 Hz, 1H), 7.43 (dd, *J* = 7.6, 1.0 Hz, 1H), 7.38–7.33 (m, 2H), 6.94 (d, *J* = 8.5 Hz, 1H), 6.85 (m, 2H), 4.29 (s, 2H), 3.74 (s, 3H).

Methyl 2-(4-((3-aminophenyl)thio)-3-nitrobenzoyl)benzoate (**C3-002a**): Yellow solid, 62 mg, 76 % yield. ^1^H-NMR (400 MHz, CDCl_3_) δ 8.51 (s, 1H), 8.10 (d, *J* = 7.8 Hz, 1H), 7.77 (d, *J* = 8.5 Hz, 1H), 7.69 (t, *J* = 7.5 Hz, 1H), 7.62 (t, *J* = 7.5 Hz, 1H), 7.36 (d, *J* = 7.4 Hz, 1H), 7.32–7.25 (m, 3H), 7.09 (t, *J* = 7.8 Hz, 1H), 7.05 (d, *J* = 8.6 Hz, 1H), 6.97 (d, *J* = 7.6 Hz, 1H), 6.89 (d, *J* = 8.2 Hz, 2H), 6.82 (d, *J* = 9.9 Hz, 2H), 6.54 (d, *J* = 7.7 Hz, 1H), 3.85 (s, 2H), 3.75 (s, 3H).

Methyl 2-(4-((4-aminophenyl)thio)-3-nitrobenzoyl)benzoate (**C3-003a**): Yellow solid, 80 mg, 98 % yield. ^1^H-NMR (400 MHz, CDCl_3_) δ 8.49 (d, *J* = 1.7 Hz, 1H), 8.10 (d, *J* = 7.8 Hz, 1H), 7.77 (dd, *J* = 8.6, 1.8 Hz, 1H), 7.68 (t, *J* = 7.4, 0.9 Hz, 1H), 7.61 (td, *J* = 7.5, 0.9 Hz, 1H), 7.35 (t, *J* = 7.6 Hz, 3H), 6.99 (d, *J* = 8.6 Hz, 1H), 6.78 (d, *J* = 8.4 Hz, 2H), 4.01 (s, 2H), 3.74 (s, 3H).

Methyl 2-(4-((2-amino-4-chlorophenyl)thio)-3-nitrobenzoyl)benzoate (**C3-004a**): Yellow solid, 70 mg, 79 % yield. ^1^H-NMR (400 MHz, CDCl_3_) δ 8.60 (s, 1H), 8.10 (d, *J* = 7.5 Hz, 1H), 7.78 (d, *J* = 8.5 Hz, 1H), 7.67 (dd, *J* = 13.5, 6.2 Hz, 1H), 7.62 (t, *J* = 6.9 Hz, 1H), 7.35 (d, *J* = 7.0 Hz, 2H), 6.93 (dd, *J* = 8.3, 1.2 Hz, 1H), 6.85 (s, 1H), 6.81 (d, *J* = 8.1 Hz, 1H), 4.39 (s, 2H), 3.74 (s, 3H).

Methyl 2-(4-((3,4-dichlorophenyl)thio)-3-nitrobenzoyl)benzoate (**C3-005a**): Yellow solid, 85 mg, 92 % yield. ^1^H-NMR (400 MHz, CDCl_3_) δ 8.55 (d, *J* = 1.6 Hz, 1H), 8.11 (d, *J* = 7.8 Hz, 1H), 7.82 (dd, *J* = 8.6, 1.7 Hz, 1H), 7.72 (d, *J* = 1.9 Hz, 1H), 7.69 (d, *J* = 6.7 Hz, 1H), 7.64 (d, *J* = 7.7 Hz, 1H), 7.61 (d, *J* = 8.3 Hz, 1H), 7.45 (dd, *J* = 8.2, 1.9 Hz, 1H), 7.37 (d, *J* = 7.4 Hz, 1H), 6.95 (d, *J* = 8.5 Hz, 1H), 3.76 (s, 3H).

#### 3.1.3. General Procedure for the Synthesis of Compound **C3-001** and Its Derivatives

The methyl esters of the title compounds (**C3-001a** and its derivatives) were hydrolyzed with 1 M NaOH in THF (1:1) at room temperature overnight. The mixture was then diluted with a small amount of water and washed twice with CH_2_Cl_2_. The aqueous solution was acidified by the addition of 2 M HCl. The precipitate was collected by filtration and washed with water to afford the titled compounds. If the compound was not pure at this stage of the procedure, it was purified by column chromatography.

2-(4-((2-Aminophenyl)thio)-3-nitrobenzoyl)benzoic Acid (**C3-001**): The title compound was prepared from the hydrolysis of ***C3-001a*** (60 mg, 0.15 mmol) in 1N NaOH (0.8 mL) and THF (0.8 mL). Yellow solid, 50 mg, 85 % yield, mp 217–219 °C. ^1^H-NMR (400 MHz, DMSO-d6) δ 13.30 (s, 1H), 8.33 (d, *J* = 1.4 Hz, 1H), 8.02 (d, *J* = 7.6 Hz, 1H), 7.82–7.72 (m, 2H), 7.69 (t, *J* = 7.2 Hz, 1H), 7.44 (d, *J* = 7.3 Hz, 1H), 7.35 (d, *J* = 7.5 Hz, 1H), 7.28 (t, *J* = 7.3 Hz, 1H), 6.90 (d, *J* = 8.6 Hz, 1H), 6.83 (d, *J* = 8.1 Hz, 1H), 6.65 (t, *J* = 7.3 Hz, 1H), 5.62 (s, 2H). ^13^C-NMR (100 MHz, DMSO-d6) δ 167.4, 151.4, 145.1, 143.1, 141.2, 137.7, 134.8, 133.3, 132.9, 132.9, 130.9, 130.5, 130.2, 127.8, 127.4, 126.1, 117.3, 115.7, 109.1. HRMS (ESI): calcd for C_20_H_13_N_2_O_5_S, (M−H)^−^ 393.0551, found 393.0547. HPLC purity: 97.00%.

2-(4-((3-Aminophenyl)thio)-3-nitrobenzoyl)benzoic Acid (**C3-002**): The title compound was prepared from the hydrolysis of **C3-002a** (60 mg, 0.15 mmol) in 1N NaOH (0.7 mL) and THF (0.7 mL). Yellow solid, 40 mg, 69 % yield, mp 123-125 °C. ^1^H-NMR (400 MHz, DMSO-d6) δ 13.31 (s, 1H), 8.31 (d, *J* = 1.1 Hz, 1H), 8.02 (d, *J* = 7.4 Hz, 1H), 7.85–7.73 (m, 2H), 7.69 (t, *J* = 7.4 Hz, 1H), 7.46 (d, *J* = 7.2 Hz, 1H), 7.20 (t, *J* = 7.7 Hz, 1H), 7.08 (d, *J* = 8.5 Hz, 1H), 6.79 (s, 1H), 6.74 (t, *J* = 8.7 Hz, 2H), 5.53 (s, 2H). ^13^C-NMR (100 MHz, DMSO-d6) δ 194.5, 167.2, 151.1, 144.8, 144.4, 140.7, 134.5, 133.6, 133.2, 131.5, 130.7, 130.4, 130.2, 129.3, 128.9, 127.9, 126.0, 122.4, 120.1, 116.5. HRMS (ESI): calcd for C_20_ H_13_N_2_O_5_S, (M−H)^−^ 393.0551, found 393.0544. HPLC purity: 95.24%.

2-(4-((4-Aminophenyl)thio)-3-nitrobenzoyl)benzoic Acid (**C3-003**): The title compound was prepared from the hydrolysis of **C3-003a** (80 mg, 0.2 mmol) in 1N NaOH (1.0 mL) and THF (1.0 mL). Yellow solid, 60 mg, 76 % yield, mp 129–131 °C. ^1^H-NMR (400 MHz, DMSO-d6) δ 13.31 (s, 1H), 8.29 (d, *J* = 1.5 Hz, 1H), 8.00 (d, *J* = 7.5 Hz, 1H), 7.80–7.71 (m, 2H), 7.67 (t, *J* = 7.3 Hz, 1H), 7.44 (d, *J* = 7.3 Hz, 1H), 7.23 (d, *J* = 8.4 Hz, 2H), 6.98 (d, *J* = 8.6 Hz, 1H), 6.69 (d, *J* = 8.5 Hz, 2H), 5.79 (s, 2H). ^13^C-NMR (100 MHz, DMSO-d6) δ 194.3, 167.3, 151.8, 146.9, 143.9, 141.1, 137.6, 134.36, 133.39, 133.1, 130.6, 130.4, 130.2, 128.4, 127.6, 125.9, 115.7, 111.8. HRMS (ESI): calcd for C_20_ H_13_N_2_O_5_S, (M−H)^−^ 393.0551, found 393.0548. HPLC purity: 99.73%.

2-(4-((2-Amino-4-chlorophenyl)thio)-3-nitrobenzoyl)benzoic Acid (**C3-004**): The title compound was prepared from the hydrolysis of **C3-004a** (70 mg, 0.16 mmol) in 1N NaOH (0.8 mL) and THF (0.8 mL). Yellow solid, 41 mg, 60 % yield, mp 118–120 °C. ^1^H-NMR (400 MHz, DMSO-d6) δ 13.32 (s, 1H), 8.33 (d, *J* = 1.6 Hz, 1H), 8.02 (d, *J* = 7.5 Hz, 1H), 7.76 (dd, *J* = 15.0, 7.8 Hz, 2H), 7.68 (t, *J* = 7.2 Hz, 1H), 7.44 (d, *J* = 7.3 Hz, 1H), 7.37 (d, *J* = 8.2 Hz, 1H), 6.92 (d, *J* = 8.5 Hz, 1H), 6.86 (d, *J* = 2.0 Hz, 1H), 6.66 (dd, *J* = 8.2, 2.1 Hz, 1H), 5.93 (s, 2H). ^13^C-NMR (100 MHz, DMSO-d6) δ 194.6, 167.3, 152.5, 145.3, 142.6, 141.0, 139.3, 137.4, 134.7, 133.6, 133.2, 130.6, 130.3, 127.9, 127.6, 126.04, 116.8, 114.6, 108.1. HRMS (ESI): calcd for C_20_H_12_ClN_2_O_5_S, (M−H)^−^ 427.0161, found 427.0152. HPLC purity: 100.00%.

2-(4-((3,4-Dichlorophenyl)thio)-3-nitrobenzoyl)benzoic Acid (**C3-005**): The title compound was prepared from the hydrolysis of **C3-005a** (80 mg, 0.17 mmol) in 1N NaOH (0.9 mL) and THF (0.9 mL). Yellow solid, 45 mg, 62 % yield, mp 247−248 °C. ^1^H-NMR (400 MHz, DMSO-d6) δ 13.34 (s, 1H), 8.37 (s, 1H), 8.02 (d, *J* = 13.0 Hz, 2H), 7.84 (d, *J* = 8.3 Hz, 1H), 7.76 (dd, *J* = 14.9, 7.7 Hz, 2H), 7.72–7.67 (m, 1H), 7.64 (d, *J* = 8.0 Hz, 1H), 7.46 (d, *J* = 6.8 Hz, 1H), 7.07 (d, *J* = 8.4 Hz, 1H). ^13^C-NMR (100 MHz, DMSO-d6) δ 194.4, 167.2, 144.8, 142.7, 140.7, 137.4, 136.1, 135.0, 134.4, 134.1, 133.3, 132.9, 130.8, 130.7, 130.5, 130.1, 129.5, 127.8, 125.8. HRMS (ESI): calcd for C_20_H_10_Cl_2_NO_5_S, (M−H)^−^ 445.9662, found 445.9651. HPLC purity: 99.73%.

### 3.2. Biology

#### 3.2.1. Bacterial Strains and Antibiotics

The following bacterial strains were used in this study for the microdilution assay: *Enterococcus faecalis* ATCC 19433, *Streptococcus pneumoniae* ATCC 49619, *Klebsiella pneumoniae* ATCC 700603, *Acinetobacter baumannii* ATCC 19606, *Pseudomonas aeruginosa* ATCC 27853, *Enterobacter cloacae* ATCC 13047, *Escherichia coli* ATCC 25922, *Streptococcus pyogenes* ATCC 19615, *Streptococcus agalactiae* ATCC 12386, *Staphylococcus epidermidis* ATCC 12228 and *Staphylococcus saprophyticus* ATCC 15305 (the American Type Culture Collection, Manassas, Virginia, United States). The antibiotic controls were purchased from Sigma-Aldrich (St. Louis, Missouri, United States). 

#### 3.2.2. Determination of Minimum Inhibitory Concentration (MIC)

The antimicrobial activity of the compounds was determined by broth microdilution according to the Clinical & Laboratory Standards Institute (CLSI) guidelines [15]. The test medium was brain heart infusion (BHI) for *S. pneumoniae*, *S. pyogenes* and *S. agalactiae*, and Mueller-Hinton (MH) broth for the rest of the organisms. Serial two-fold dilutions were performed for the tested chemicals starting from 256 μg/mL to 0.5 μg/mL, and the bacterial cell inoculum was adjusted to approximately 1.5 × 10^6^ CFU per mL. Results were taken after 16–20 hrs of incubation at 37 °C (with 5% CO_2_ supplementation for the *Streptococcus* spp.). The MIC was defined as the lowest concentration of antibiotic with no visible growth. Experiments were performed in duplicate. 

#### 3.2.3. Protein-Protein Interaction Inhibition Assay

Previously established protocols were used for inhibitor testing with modifications [14]. Vectors were made in which *B. subtilis* RpoC clamp-helix (CH)-domain (220–315 aa) was tagged with SmBiT NanoLuc fragment at its N-terminal (pCU252) and full-length SigA tagged with LgBiT NanoLuc fragment at its C-terminal (pCU251) [14]. Protein overproduction and purification were performed as detailed previously [16]. 40 μL of purified C-SmBiT-CH (0.125 μM in PBS) was added to 96-well plates and then mixed with 20 μL compound (50 μM in PBS). The mixture was incubated for 10 min at 37°C. 40 μL N-LgBiT-SigA (0.125 μM in PBS) was then added to each well, followed by incubation for 10 min at 37 °C. The final concentration of the compounds was at 10 μM. After the final incubation step, equal volume of Nano-Glo^®^ Luciferase Assay Substrate (Promega, Madison, Wisconsin, United States) was added to the reaction mixture. Luminescence emitted was measured using a Victor X3 Multilabel plate reader (Waltham, Massachusetts, United States). Experiment was performed in triplicate. Technical repeats were taken to ensure consistent results were obtained.

#### 3.2.4. Time-Kill Kinetics

*S. pneumoniae* cells were suspended to ~1.5 × 10^6^ CFU/mL at log phase in BHI medium with compounds at various concentrations. As an untreated control, bacteria were incubated in BHI medium without compounds. The cultures were grown at 37 °C with shaking at 200 rpm supplemented with 5% CO_2_, where 20 μL samples were taken at defined time points (0, 2, 4 and 6 h) for each treatment group, followed by 10-fold serial dilutions. 5 μL sample were taken from each dilution and spotted on blood agar plate. After overnight incubation at 37 °C with 5% CO_2_, the number of viable bacteria in each sample was counted and expressed as CFU/mL. The experiment was performed in triplicate. Technical repeats were taken to ensure consistent results were obtained.

#### 3.2.5. Assessment of ATP Production

*S. pneumoniae* cells were suspended to ~1.5 × 10^6^ CFU/mL at log phase in BHI medium with compounds at various concentrations. As an untreated control, bacteria were incubated in BHI medium without compounds. The cultures were grown at 37 °C with shaking at 200 rpm and at 5% CO_2_, where 100 μL samples were taken at defined time points (0, 2, 4 and 6 h) for each treatment group. The ATP production was measured using the BacTiter-Glo™ Microbial Cell Viability Assay Kit (Promega, Madison, Wisconsin, United States) according to the manufacturer’s instructions. Experiment was performed in triplicate. Technical repeats were taken to ensure consistent results were obtained.

#### 3.2.6. *S. pneumoniae* Toxin Secretion

*S. pneumoniae* cells were grown overnight without agitation on round-bottomed 96-well plates in the presence of serially diluted concentrations of compounds. The antibiotics were also added at serial two-fold dilutions starting from 4 μg/mL to 0.003 μg/mL. After 16–20 h incubation, each corresponding ½ × and ¼ × MIC values were determined. The cultures were then resuspended with their OD_595_ readings measured and the plates were centrifuged at 3000× *g* for 3 min. The supernatants from the cultures which were challenged with ½ × and ¼ × MIC of the test compounds, antibiotics and drug-free controls were transferred to a fresh plate and were subsequently used for Western blot assay.

#### 3.2.7. Western Blot

Samples were separated in 10% polyacrylamide gels at 150 V for 1 h, before being transferred to a PVDF membrane at 110 V for 1 h. The membrane was then blocked with 5% non-fat milk in TBST buffer for 1 h, incubated overnight with 1:1000 rabbit polyclonal anti-pneumolysin primary antibody (ab71811, abcam, Cambridge, United Kingdom) at 4 °C with agitation, followed by 1 hr incubation with 1:5000 goat polyclonal anti-rabbit HRP-conjugated secondary antibody (ab97051, abcam, Cambridge, United Kingdom) at room temperature with agitation, complete with TBST-washing cycles prior and after. Blots were incubated with Bio-Rad Clarity^TM^ Western ECL Substrates and the resulting bands were visualized in a Bio-Rad ChemiDoc^TM^ Touch imaging system in Chemiluminescence mode (Bio-Rad, Hercules, California, United States). The experiment was performed in triplicate. Technical repeats were taken to ensure consistent results were obtained.

#### 3.2.8. Cytotoxicity Assay

Human cell lines A549 lung carcinoma and HepG2 hepatoblastoma were used in this study. The cells were seeded at 2.5 × 10^5^ per well. After 24 h incubation, the tested compounds were added in a 2-fold serial dilution ranging from 1562 μg/mL to 50 μg/mL. The plates were then incubated at 37 °C. At 48 h and 72 h after adding the compound, the MTT assay was performed as described previously [25]. Cisplatin was used as the positive control and DMSO as the negative control.

### 3.3. Molecular Modelling

Modelling was performed using UCSF chimera [26]. *E. coli* RNAP holoenzyme crystal structure (PDB: 4LJZ) was employed [27]. Images were made with UCSF chimera [26]. The pharmacophore model was generated with Discovery Studio 2016 (Biovia, San Diego, California, United States).

### 3.4. Data and Statistical Analysis

Technical repeats were taken for the biochemical assays to ensure reproducibility. One-way ANOVA was used to measure the statistical significance. The data was presented in GraphPad Prism style: *p* ≤ 0.05 (*), ≤ 0.01 (**), ≤ 0.001 (***), ≤ 0.0001 (****).

## 4. Conclusions

Herein, we report the discovery and evaluation of novel analogues of inhibitors against bacterial RNAP and σ factor interaction. By the pharmacophore model-based rational design followed by synthesis of analogues, we were able to obtain a **C3** derivative with greater inhibitory activity by the additional interaction with RNAP CH N294 through hydrophobic interaction with a chloride group, and significantly improved antibacterial activity against Gram-positive pathogens. The latter may be attributed to the greater cell permeability which can be reflected by an elevated ClogP value calculated by Discovery Studio 2016 (Biovia, San Diego, California, United States) as shown in Table 2. The logP values represent the logarithm of the ratio of compound solubility in octanol and water. As a result, the molecule with high logP values may display an unprecedented bacterial cell permeability. The mechanism of the **C3** derivatives against the PPI between RNAP CH and σ was confirmed at the molecular level by an in vitro luciferase complementation assay. The compound with the best antimicrobial activity, **C3-005**, has been shown to be bactericidal at higher concentrations and able to suppress ATP production in *S. pneumoniae* cells, like rifampicin as a bacterial transcription inhibitor. It also showed more suppression of the *S. pneumoniae* virulence factor pneumolysin secretion than rifampicin. Since bacterial transcription is a proven but under-utilized target for antibiotics, our approach may lead the way to a valid platform for novel antimicrobial discovery. On top of their therapeutic potential, the compounds described in this report could also complement the development of chemical probes to study the regulation of transcription by σ factors.

## Figures and Tables

**Table 1:** Antimicrobial activity of **C3** and derivatives.

Compound	EFAE	SAUR^a^	SAUR^b^	SPNE	KPNE	ABAU	PAER	ECLO	ECOL
**C3**	>256	>256	>256	256	>256	>256	>256	>256	>256
**C3-002**	256	256	256	64	>256	>256	>256	>256	>256
**C3-003**	>256	>256	>256	128	>256	>256	>256	>256	>256
**C3-004**	256	128	256	64	>256	>256	>256	>256	>256
**C3-005**	32	16	16	8	>256	>256	>256	>256	>256
**VAN**	1	1	0.5	0.25	>64	>64	>64	>64	>64
**RIF**	4	0.063	0.063	0.063	32	4	32	≥64	64

EFAE: *Enterococcus faecalis* ATCC 19433, SAUR^a^: *S. aureus* ATCC 25923, SAUR^b^: *S. aureus* ATCC 29213, SPNE: *Streptococcus pneumoniae* ATCC 49619, ABAU: *Acinetobacter baumannii* ATCC 19606, PAER: *Pseudomonas aeruginosa* ATCC 27853, ECLO: *Enterobacter cloacae* ATCC 13047, ECOL: *E. coli* ATCC 25922, VAN: vancomycin, RIF: rifampicin.

**Table 2:** Antimicrobial and inhibitory activities of **C3** derivatives.

Compound	R	MIC ^a^ (μg/mL)	% Inhibition ^b^	ClogP
**C3-001**	2-NH_2_	256	62.2 ± 2.6	3.39
**C3-002**	3-NH_2_	64	81.3 ± 4.9	3.39
**C3-003**	4-NH_2_	128	69.1 ± 10.7	3.39
**C3-004**	2-NH_2_, 4-Cl	64	60.5 ± 16.5	4.39
**C3-005**	3-Cl, 4-Cl	8	81.7 ± 0.9	5.94
**Vancomycin**	N.A.	0.25	N.D.	N.D.
**Rifampicin**	N.A.	0.0625	N.D.	N.D.

^a^ The MIC values against *S. pneumoniae* ATCC 49619; ^b^ the percentages of inhibition of **C3** and derivatives at 10 μM against the PPI between RNAP CH-σ at 0.05 μM.

**Table 3:** Antimicrobial activity (MIC μg/mL) of **C3-005** against representative Gram-positive bacterial pathogens.

Compound	MIC (μg/mL)			
	SPYO	SAGA	SEPI	SSAP
**C3-005**	16	16	16	32
**VAN**	0.5	1	2	1
**RIF**	0.031	0.031	≤0.063	≤0.063

SPYO: *Streptococcus pyogenes* ATCC 19615, SAGA: *Streptococcus agalactiae* ATCC 12386, SEPI: *Staphylococcus epidermidis* ATCC 12228, SSAP: *Staphylococcus saprophyticus* ATCC 15305, VAN: vancomycin, RIF: rifampicin.

**Table 4:** Cytotoxicity of **C3-005** against Human HepG2 and A549 cell lines.

Compound	CC_50_ (μM)	
	HepG2	A549
**C3-005**	63.82 ± 9.32	76.65 ± 11.37
**cisplatin**	6.81 ± 0.61	7.70 ± 0.58

## Supplementary Materials

Figure S1: IC_50_ measurement of **C3** against the protein-protein interaction between RNAP CH-σ, Table S1: The luminescence data of **C3** inhibiting the RNAP CH–σ protein-protein interaction, ^1^H- & ^13^C-NMR: **C3-001**, **C3-002**, **C3-003**, **C3-004**, **C3-005**, and HPLC spectra: **C3-001**, **C3-002**, **C3-003**, **C3-004**, **C3-005** (PDF).
