# Supplementary material for: First-In-Class Inhibitors Targeting the Interaction between Bacterial RNA Polymerase and Sigma Initiation Factor Affect the Viability and Toxin Release of Streptococcus pneumoniae
Source: Molecules. 2019 Aug 9;24(16):2902. doi: 10.3390/molecules24162902 (PMC6719014; doi:10.3390/molecules24162902)
Supplement: Supplementary file 1 [file molecules-24-02902-s001.pdf]

First-In-Class Inhibitors Targeting the Interaction between Bacterial RNA Polymerase and Sigma Initiation Factor Affect the Viability and Toxin Release of *Streptococcus pneumoniae*

Jiqing Ye [1,†] Adrian Jun Chu [2,†] Lin Lin [2] Xiao Yang [2,*] and Cong Ma [1,*]

[1] State Key Laboratory of Chemical Biology and Drug Discovery, Department of Applied Biology and Chemical Technology, The Hong Kong Polytechnic University, Kowloon, Hong Kong

[2] Department of Microbiology, The Chinese University of Hong Kong, Prince of Wales Hospital, Shatin, Hong Kong

* Correspondence: cong.ma@polyu.edu.hk (C.M.); xiaoyang@cuhk.edu.hk (X.Y.)

† These authors contributed equally to this work.

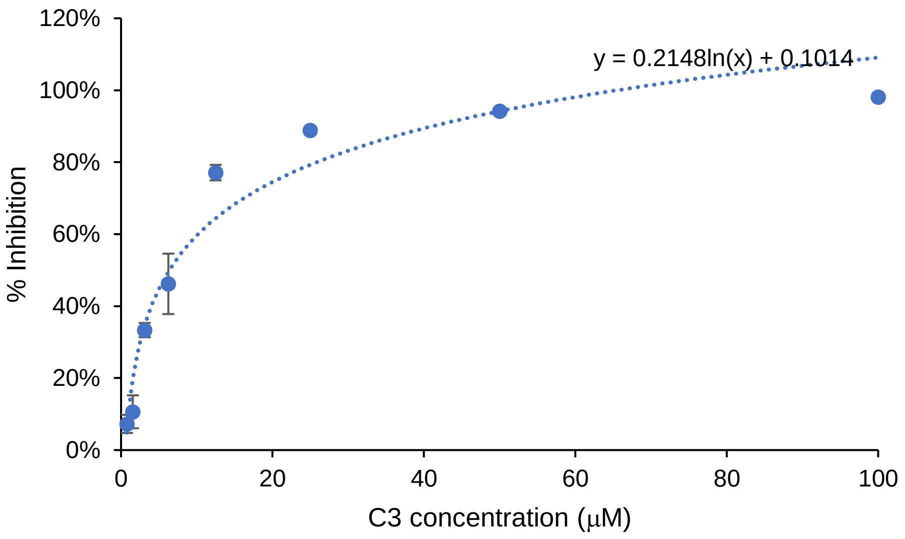

Figure 1. IC_{50} measurement of C3 against the protein-protein interaction between RNAP CH-σ at 0.05 μM.

Table S1. The luminescence data of C3 inhibiting the RNAP CH – σ protein-protein interaction.

Triplicate A	Triplicate B	Triplicate C	Average	% Inhibition	% SE[a]	C3 concentration (μM)
25819	33863	27577	29086	100%	8.39%	0
632	614	434	560	98.07%	0.22%	100
1848	1776	1422	1682	94.22%	0.45%	50
3074	3836	2850	3253	88.81%	1.03%	25
6800	7662	5510	6657	77.11%	2.15%	12.5
14861	20218	11868	15649	46.20%	8.40%	6.25
18575	20504	19085	19388	33.34%	1.98%	3.125
27429	27207	23336	25991	10.64%	4.57%	1.5625
25793	28308	26815	26972	7.27%	2.51%	0.78125

[a] Standard error

^{1}H NMR and ^{13}C NMR spectra.

C3-001

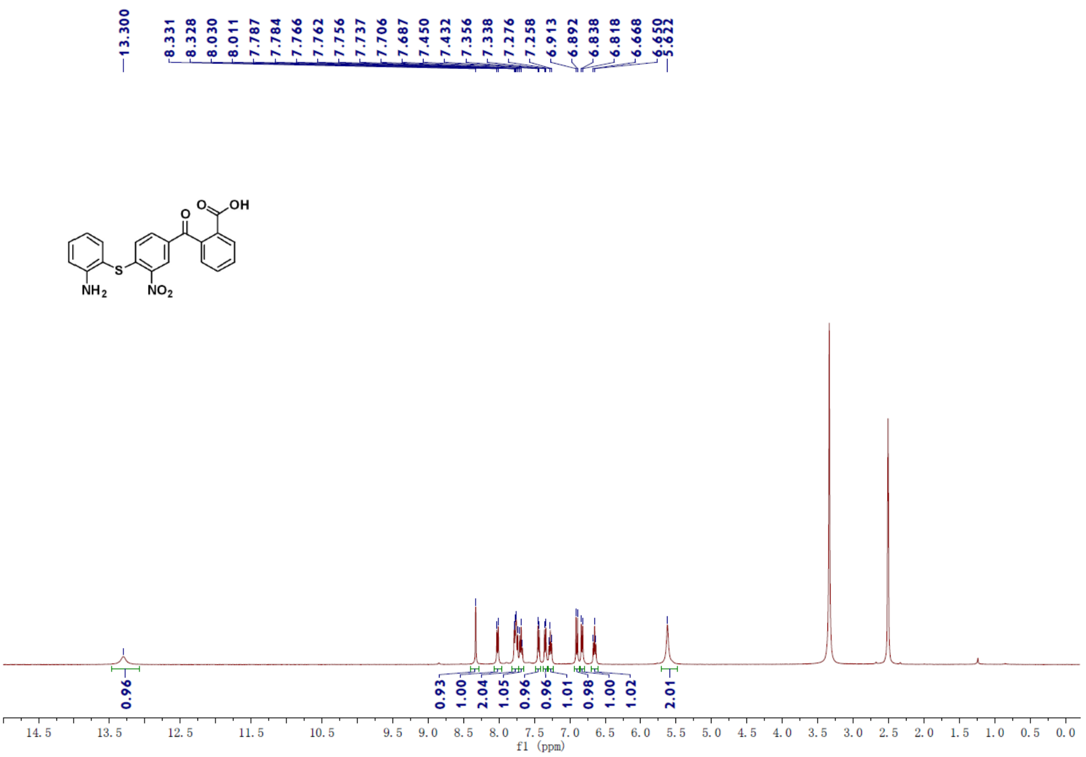

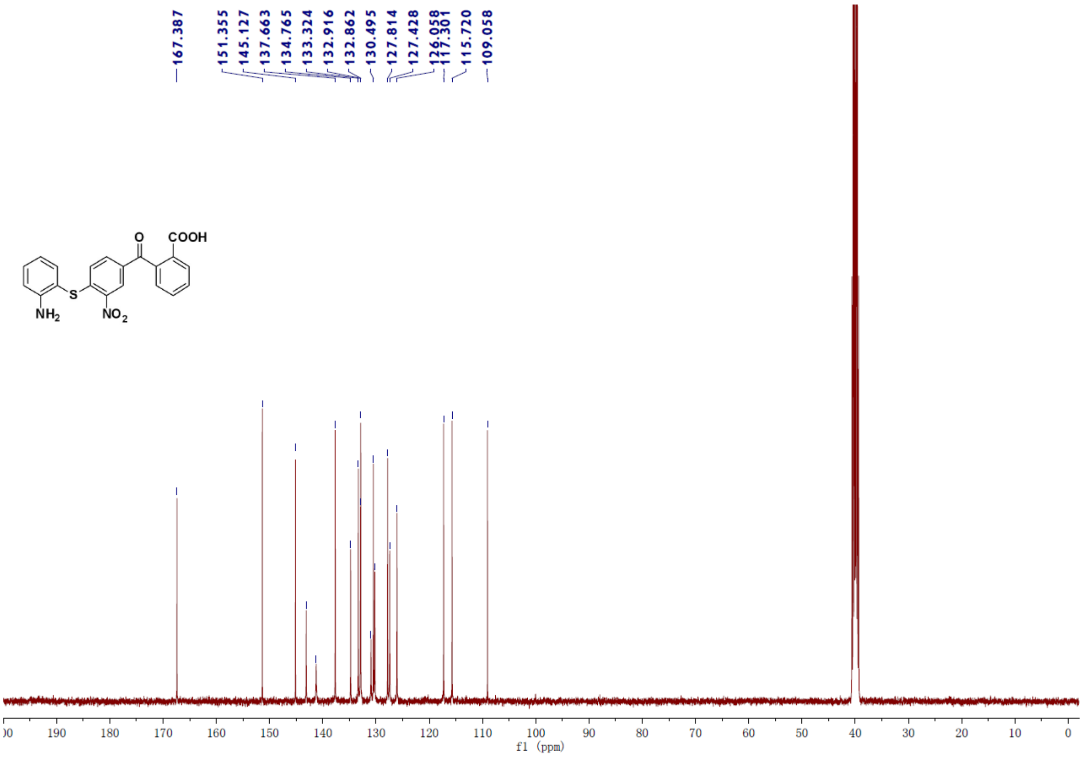

C3-002

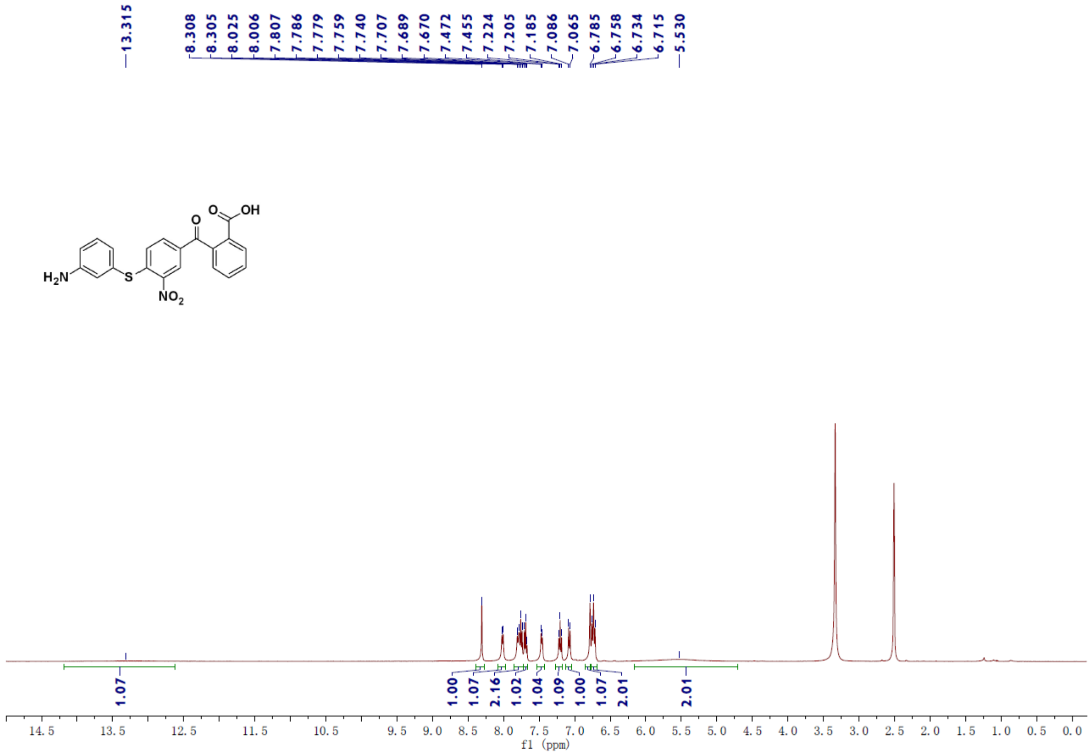

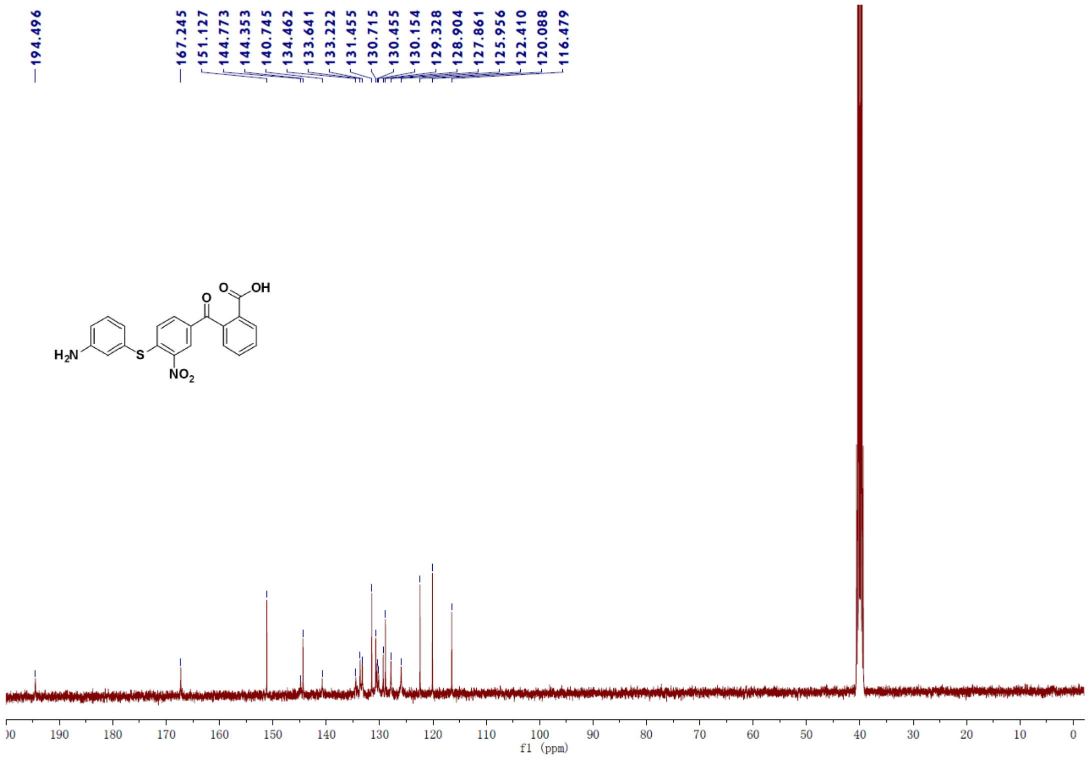

C3-003

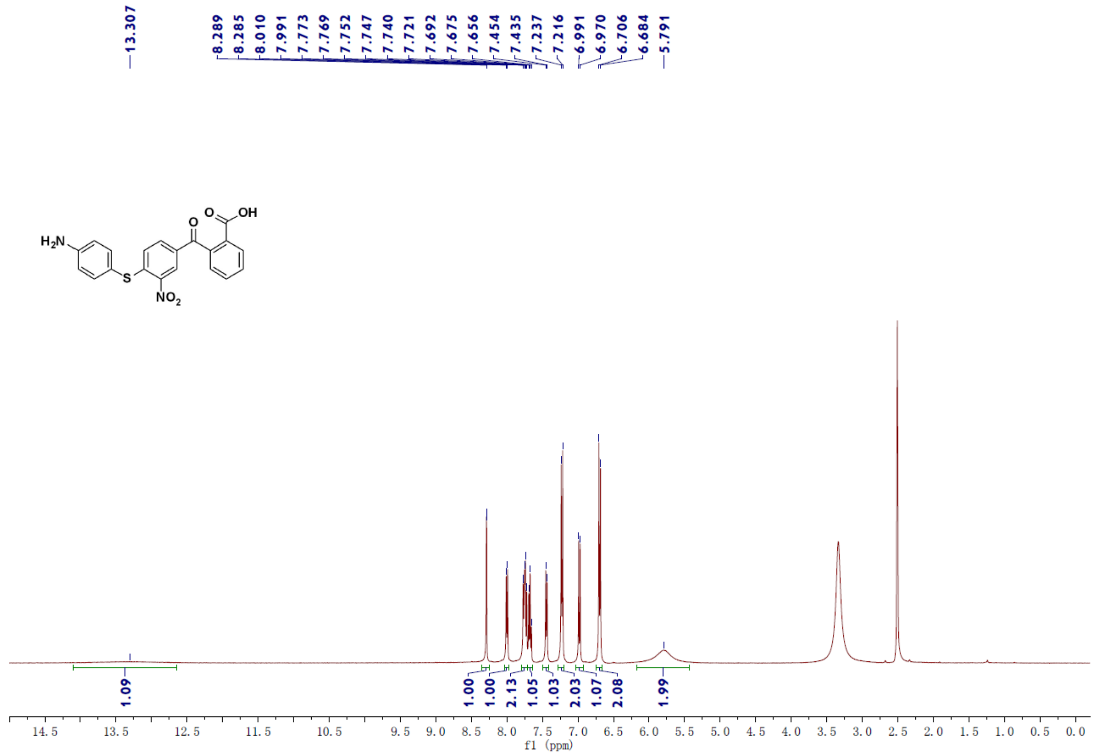
13.307
8.289
8.285
8.010
7.991
7.773
7.769
7.752
7.747
7.740
7.721
7.692
7.675
7.656
7.454
7.435
7.237
7.216
6.991
6.970
6.706
6.684
5.791
O
OH
O
H2N
S
NO2
1.09
1.00
1.00
2.13
1.05
1.03
2.03
1.07
2.08
1.99
f1 (ppm)

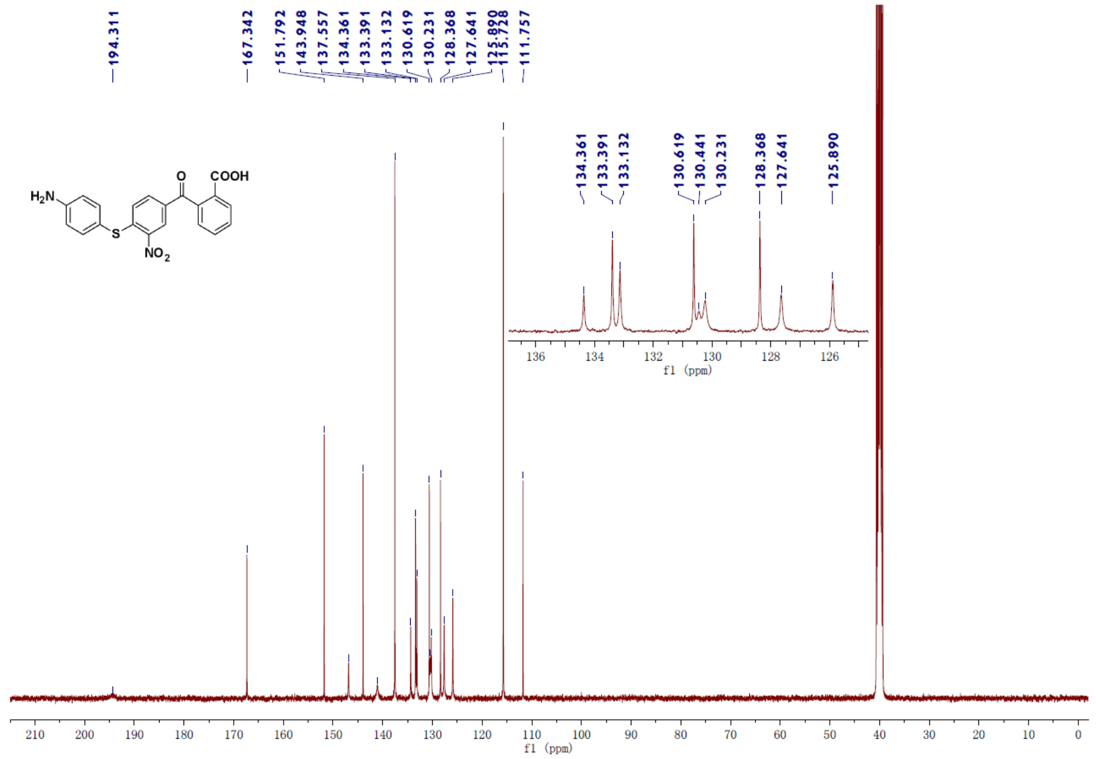
194.311
167.342
151.792
143.948
137.557
134.361
133.391
133.132
130.619
130.231
128.368
127.641
125.890
115.728
111.757
O
COOH
H2N
S
NO2
134.361
133.391
133.132
130.619
130.441
130.231
128.368
127.641
125.890
f1 (ppm)
f1 (ppm)

C3-004

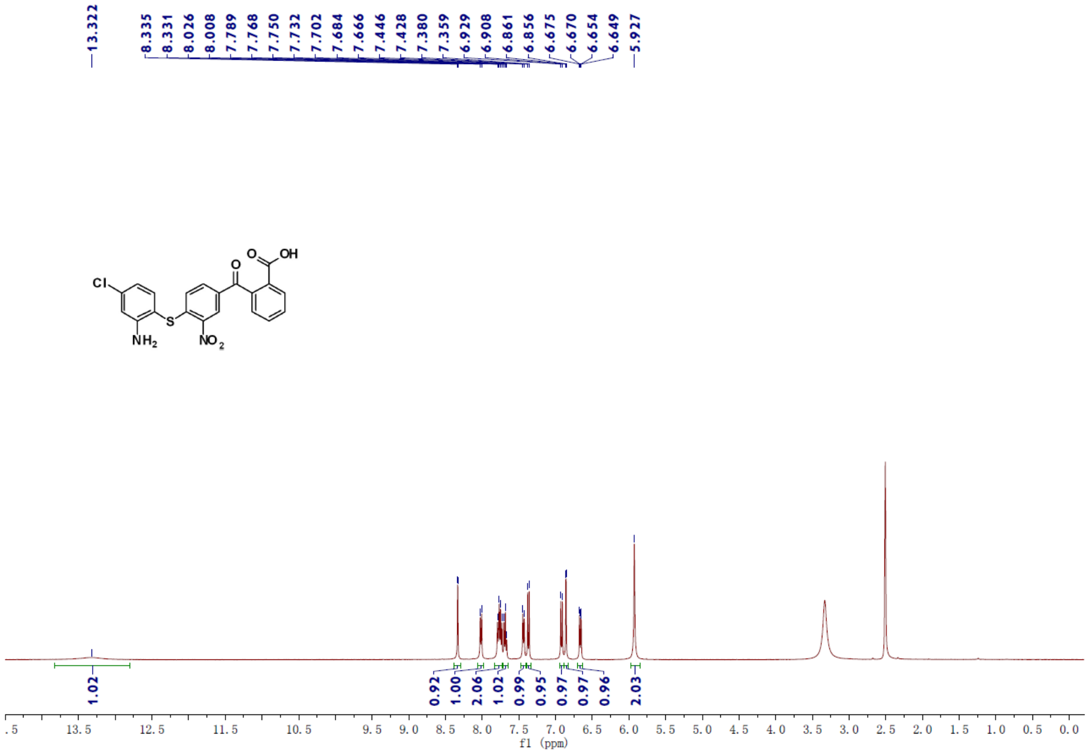

13.322
8.335
8.331
8.026
8.008
7.789
7.768
7.750
7.732
7.702
7.684
7.666
7.446
7.428
7.380
7.359
6.929
6.908
6.861
6.856
6.675
6.670
6.654
6.649
5.927
Cl
NH2
S
NO2
O
O
OH
1.02
0.92
1.00
2.06
1.02
0.99
0.95
0.97
0.97
0.96
2.03
f1 (ppm)

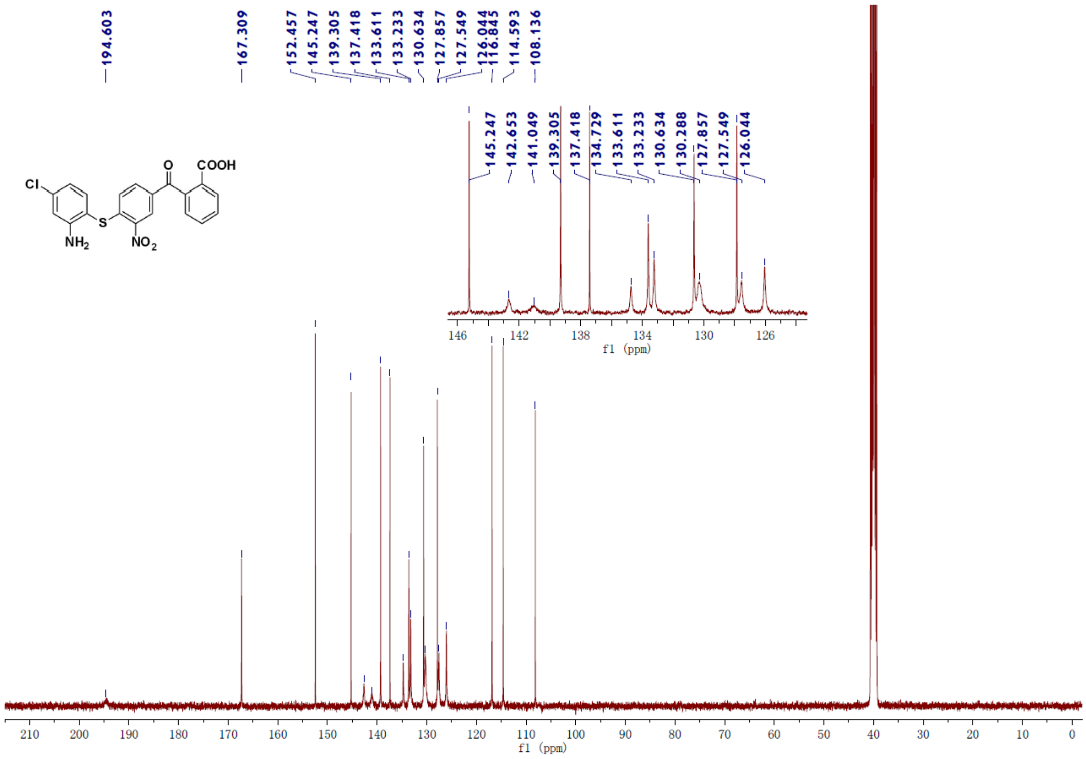

194.603
167.309
152.457
145.247
139.305
137.418
133.611
133.233
130.634
127.857
127.549
126.044
116.845
114.593
108.136
145.247
142.653
141.049
139.305
137.418
134.729
133.611
133.233
130.634
130.288
127.857
127.549
126.044
Cl
NH2
S
NO2
O
COOH
f1 (ppm)

C3-005

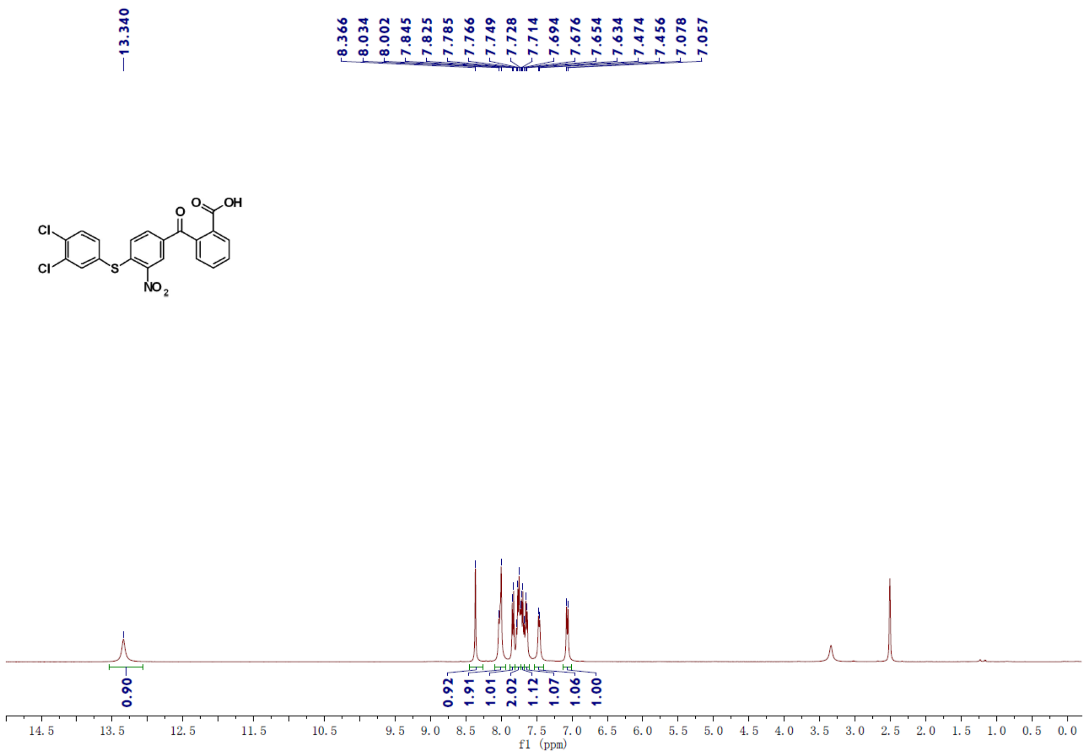
13.340
8.366
8.034
8.002
7.845
7.825
7.785
7.766
7.749
7.728
7.714
7.694
7.676
7.654
7.634
7.474
7.456
7.078
7.057
Cl
Cl
S
O
OH
NO2
0.90
0.92
1.91
1.01
2.02
1.12
1.07
1.06
1.00
f1 (ppm)

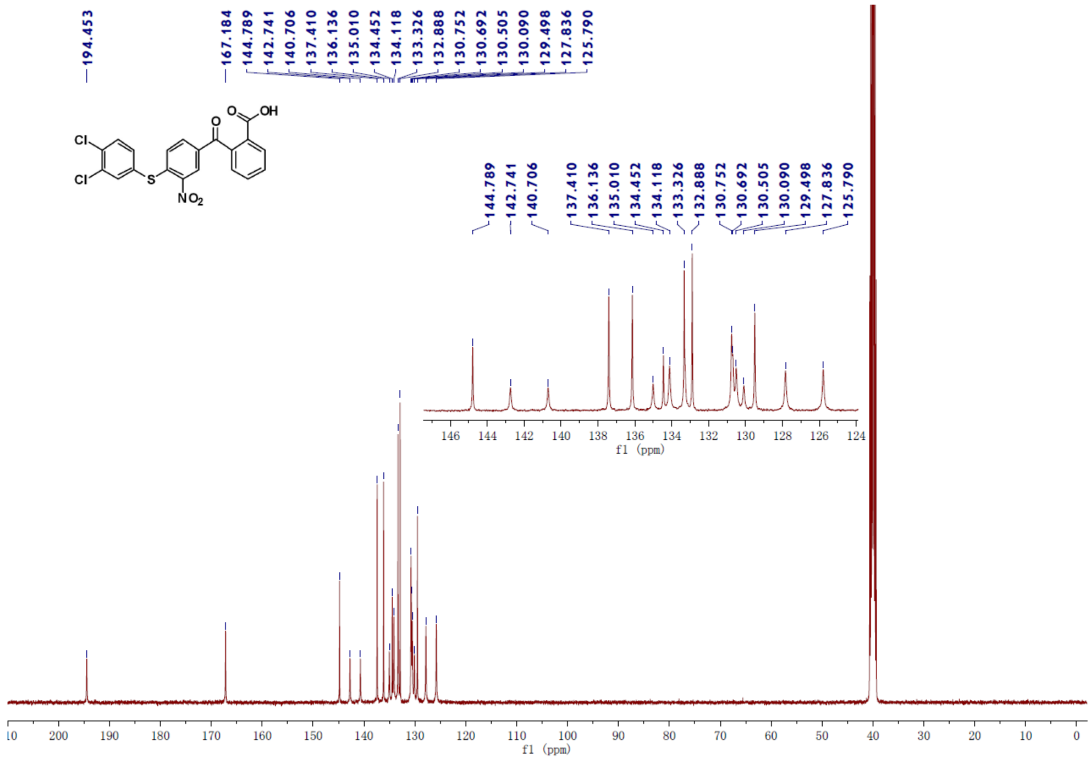
194.453
167.184
144.789
142.741
140.706
137.410
136.136
135.010
134.452
134.118
133.326
132.888
130.752
130.692
130.505
130.090
129.498
127.836
125.790
Cl
Cl
S
O
OH
NO2
f1 (ppm)

HPLC spectra

HPLC analysis of final compounds

System: Agilent 1260 Infinity

Mobile phase: Mobile phase A: MeCN; mobile phase B: H_2O

Detector: MWD at 254 nm

Column: Agilent ZORBAX Eclipse plus C18 (4.6 × 100 mm, 5 μm)

Flow rate: 1.000 mL/min

Using gradients elution.

C3-001

Data File C:\CHEM32\1\DATA\YEJIQING\20190110-5.D
Sample Name: 20190110-5

===

```
Acq. Operator   : QYY
Acq. Instrument : Instrument 1                Location : Vial 3
Injection Date  : 1/10/2019 5:45:01 PM
Acq. Method     : C:\CHEM32\1\METHODS\YEJIQING.M
Last changed    : 1/10/2019 5:44:11 PM by QYY
Analysis Method : C:\CHEM32\1\METHODS\WU HAICUI.M
Last changed    : 1/11/2019 8:19:24 PM by yejiqing
                  (modified after loading)
Sample Info     : C3-18-001
```

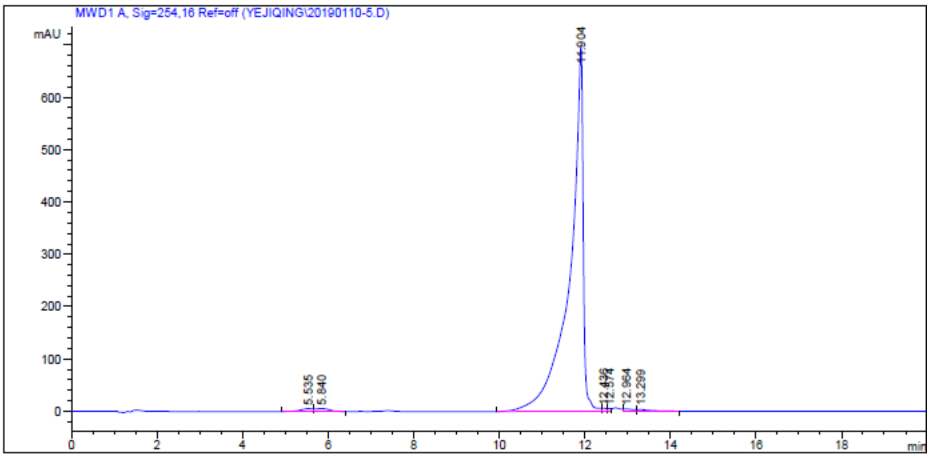

===

Area Percent Report

===

```
Sorted By             :      Signal
Multiplier            :      1.0000
Dilution              :      1.0000
Sample Amount         :      1.00000  [ng/ul]   (not used in calc.)
Use Multiplier & Dilution Factor with ISTDs
```

Signal 1: MWD1 A, Sig=254,16 Ref=off

Peak #	RetTime [min]	Type	Width [min]	Area [mAU*s]	Height [mAU]	Area %
1	5.535	BV	0.2844	114.39557	5.89585	0.7429
2	5.840	VB	0.3480	131.97195	5.87640	0.8570

Peak #	RetTime [min]	Type	Width [min]	Area [mAU*s]	Height [mAU]	Area %
3	11.904	BV	0.2759	1.49381e4	697.45142	97.0034
4	12.436	VV	0.1152	46.18891	5.65991	0.2999
5	12.574	VV	0.0730	27.20704	5.20154	0.1767
6	12.964	VV	0.2077	73.63541	4.62694	0.4782
7	13.299	VB	0.2561	68.06255	3.39128	0.4420
Totals :				1.53995e4	728.10334	

===

*** End of Report ***

C3-002

Data File C:\CHEM32\1\DATA\YEJIQING2\2018090371.D
Sample Name: 2018090371

===

```
Acq. Operator   : YeJiqing
Acq. Instrument : Instrument 1                   Location : Vial 1
Injection Date  : 9/6/2018 11:43:04 AM
Acq. Method     : C:\CHEM32\1\METHODS\YEJIQING.M
Last changed    : 9/6/2018 11:42:43 AM by YeJiqing
Analysis Method : C:\CHEM32\1\METHODS\YEJIQING.M
Last changed    : 12/12/2018 9:27:24 PM by SXY
                  (modified after loading)
Sample Info     : C3-18-031
```

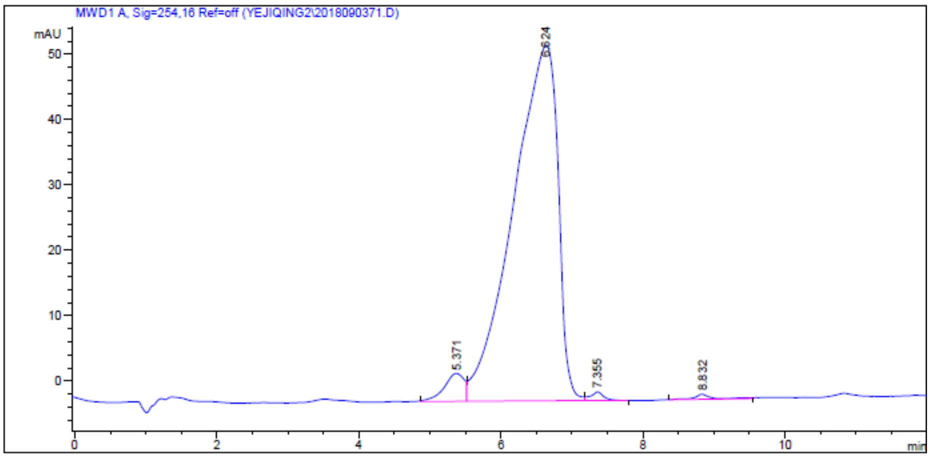

===

Area Percent Report

===

```
Sorted By             :      Signal
Multiplier            :      1.0000
Dilution              :      1.0000
Sample Amount         :      1.00000  [ng/ul]   (not used in calc.)
Use Multiplier & Dilution Factor with ISTDs
```

Signal 1: MWD1 A, Sig=254,16 Ref=off

Peak #	RetTime [min]	Type	Width [min]	Area [mAU*s]	Height [mAU]	Area %
1	5.371	BV	0.2961	85.72115	4.30946	3.4111
2	6.624	VV	0.6259	2393.43896	54.33510	95.2416

Peak #	RetTime [min]	Type	Width [min]	Area [mAU*s]	Height [mAU]	Area %
3	7.355	VB	0.1956	17.92930	1.34427	0.7135
4	8.832	BB	0.2822	15.92837	7.93498e-1	0.6338

Totals : 2513.01778   60.78234

===

*** End of Report ***

C3-003

Data File C:\CHEM32\1\DATA\YEJIQING2\2018090379.D
Sample Name: 2018090379

===

Acq. Operator : YeJiqing
Acq. Instrument : Instrument 1 Location : Vial 1
Injection Date : 9/6/2018 3:58:52 PM
Acq. Method : C:\CHEM32\1\METHODS\YEJIQING.M
Last changed : 9/6/2018 3:56:38 PM by YeJiqing
Analysis Method : C:\CHEM32\1\METHODS\YEJIQING.M
Last changed : 12/12/2018 9:39:33 PM by SXY
(modified after loading)
Sample Info : C3-18-032

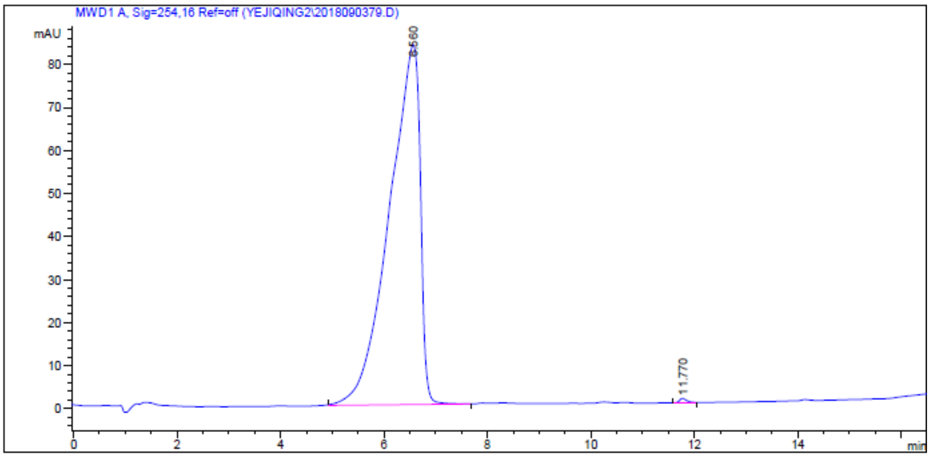

===

Area Percent Report

===

Sorted By : Signal
Multiplier : 1.0000
Dilution : 1.0000
Sample Amount : 1.00000 [ng/ul] (not used in calc.)
Use Multiplier & Dilution Factor with ISTDs

Signal 1: MWD1 A, Sig=254,16 Ref=off

Peak #	RetTime [min]	Type	Width [min]	Area [mAU*s]	Height [mAU]	Area %
1	6.560	BB	0.5863	3484.63843	83.59244	99.7268
2	11.770	BB	0.1470	9.54592	1.00294	0.2732

Data File C:\CHEM32\1\DATA\YEJIQING2\2018090379.D
Sample Name: 2018090379

Peak #	RetTime [min]	Type	Width [min]	Area [mAU*s]	Height [mAU]	Area %
Totals :				3494.18435	84.59538	

===

*** End of Report ***

C3-004

Data File C:\CHEM32\1\DATA\YEJIQING\2018090339.D
Sample Name: 2018090339

===

Acq. Operator : YeJiqing
Acq. Instrument : Instrument 1 Location : Vial 1
Injection Date : 9/4/2018 7:38:29 PM
Acq. Method : C:\CHEM32\1\METHODS\YEJIQING.M
Last changed : 9/4/2018 7:36:05 PM by YeJiqing
Analysis Method : C:\CHEM32\1\METHODS\YEJIQING.M
Last changed : 9/4/2018 8:20:16 PM by YeJiqing
(modified after loading)
Sample Info : C3-18-063

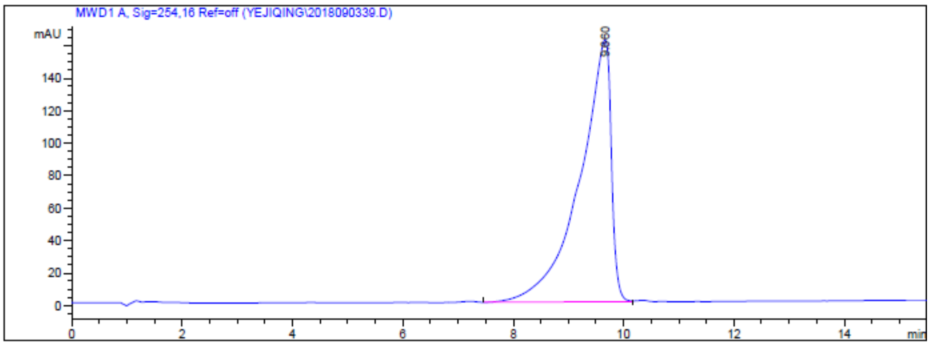

===
Area Percent Report
===

Sorted By : Signal
Multiplier : 1.0000
Dilution : 1.0000
Sample Amount : 1.00000 [ng/ul] (not used in calc.)
Use Multiplier & Dilution Factor with ISTDs

Signal 1: MWD1 A, Sig=254,16 Ref=off

Peak #	RetTime [min]	Type	Width [min]	Area [mAU*s]	Height [mAU]	Area %
1	9.660	VV	0.5424	6398.52100	161.43578	100.0000

Totals : 6398.52100 161.43578

===
*** End of Report ***

C3-005

Data File C:\CHEM32\1\DATA\YEJIQING2\2018090358.D
Sample Name: 2018090358

===

```
Acq. Operator   : YeJiqing
Acq. Instrument : Instrument 1                    Location : Vial 1
Injection Date  : 9/5/2018 2:41:28 PM
Acq. Method     : C:\CHEM32\1\METHODS\YEJIQING.M
Last changed    : 9/5/2018 2:40:21 PM by YeJiqing
Analysis Method : C:\CHEM32\1\METHODS\YEJIQING.M
Last changed    : 12/12/2018 9:15:23 PM by SXY
                  (modified after loading)
Sample Info     : C3-18-005H
```

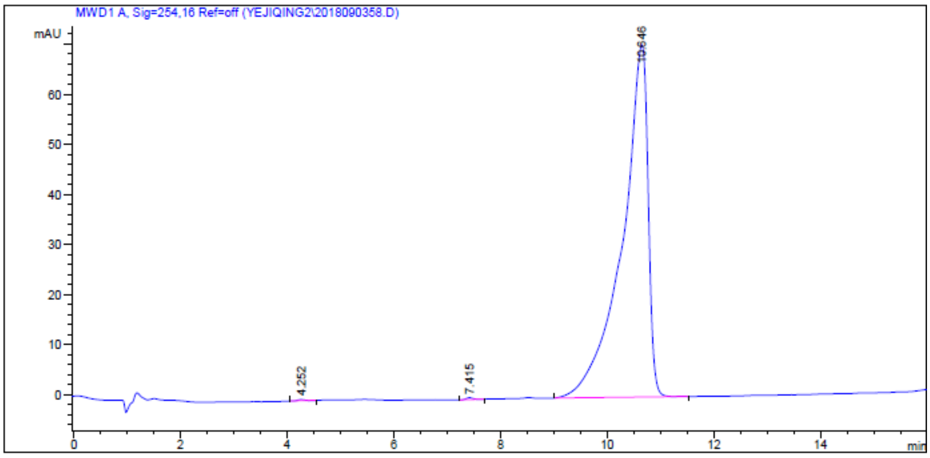

===

Area Percent Report

===

```
Sorted By             :      Signal
Multiplier            :      1.0000
Dilution              :      1.0000
Sample Amount         :      1.00000  [ng/ul]   (not used in calc.)
Use Multiplier & Dilution Factor with ISTDs
```

Signal 1: MWD1 A, Sig=254,16 Ref=off

Peak #	RetTime [min]	Type	Width [min]	Area [mAU*s]	Height [mAU]	Area %
1	4.252	BB	0.1669	2.47320	2.31038e-1	0.1045
2	7.415	BB	0.1459	3.93987	4.03477e-1	0.1665

Peak #	RetTime [min]	Type	Width [min]	Area [mAU*s]	Height [mAU]	Area %
3	10.646	BB	0.4735	2360.16724	70.33060	99.7290
Totals :				2366.58031	70.96512	

===

*** End of Report ***
